# Misclassification bias in chronic disease case ascertainment algorithms: a reclassification approach

**DOI:** 10.24095/hpcdp.46.5.02

**Published:** 2026-05

**Authors:** Naomi C. Hamm, Ruth Ann Marrie, Depeng Jiang, Pourang Irani, Lisa M. Lix

**Affiliations:** 1 College of Community and Global Health, Max Rady College of Medicine, Rady Faculty of Health Sciences, University of Manitoba, Winnipeg, Manitoba, Canada; 2 Department of Medicine, Faculty of Medicine, Dalhousie University, Halifax, Nova Scotia, Canada; 3 Department of Computer Science, Mathematics, Physics and Statistics, Irving K. Barber Faculty of Science, University of British Columbia, Kelowna, British Columbia, Canada

**Keywords:** misclassification, prevalence, algorithm, administrative health data, bias

## Abstract

**Introduction:**

Use of administrative health data to identify chronic disease cases can cause misclassification bias. Reclassification-based exit rules may reduce misclassification bias.

**Methods:**

Manitoban administrative health data (1995–2022) were used to ascertain multiple sclerosis (MS) and “juvenile diabetes” (JD) prevalence. We constructed multivariable logistic regression model-based algorithms and used a model-predicted probability exit rule to reclassify JD and MS case status annually. Sensitivity, specificity, positive predictive value (PPV), negative predictive value (NPV) and reclassification rates were estimated. Linear regression tested for differences in prevalence estimates for the model-based algorithm with an exit rule and an existing Canadian Chronic Disease Surveillance System (CCDSS) algorithm without an exit rule.

**Results:**

The MS cohort included 60228 individuals (608 cases, 59620 non-cases) and the JD cohort 44125 individuals (2506 cases, 41619 non-cases). Model-based algorithm sensitivity was 0.62 to 0.85 for MS and 0.87 to 0.95 for JD. PPV for MS was 0.21 to 0.60 and for JD was 0.92 to 0.95. Specificity and NPV were consistently high (0.98–1.00). Non-cases were frequently misclassified; reclassification rates for non-cases were higher than for cases for MS (0.22–0.33 vs. 0.14–0.28) and JD (0.18–0.65 vs. 0.13–0.15). The model-based algorithm with an exit rule for MS, but not for JD, had a slower increase in prevalence than the CCDSS algorithm.

**Conclusion:**

Case ascertainment algorithms with an exit rule can address misclassification bias when estimating chronic disease prevalence using administrative health data. Improvements are disease dependent.

**Highlights**

- The performance of the algorithms that identify cases of multiple sclerosis in individuals 20 years and older and of diabetes (both type 1 and type 2) in individuals 18 years and younger in administrative health data was better when using health care covariates based on a higher number of years.
- An exit rule that uses probabilities to reclassify case status annually found that non-cases had a higher reclassification rate than cases.
- Prevalence trends for multiple sclerosis obtained using a model-based algorithm with an exit rule had a slower increase than the current algorithm used by the Canadian Chronic Disease Surveillance System.

## Introduction

Administrative health data, such as physician billing claims and hospital discharge records, are frequently used to estimate prevalence and incidence of chronic diseases in the entire population. These estimates are obtained by applying a case ascertainment algorithm to the data; ideally this algorithm has been validated in a population with a known disease status.^1^ Algorithm validation provides estimates of sensitivity, specificity, positive predictive value (PPV) and negative predictive value (NPV) that are used by researchers and policy makers to assess the magnitude of potential bias in estimates of disease prevalence and incidence.

Misclassification of disease cases in case ascertainment algorithms for administrative health data is common.^2^ It can occur due to diagnosis coding errors, the use of nonspecific medication codes (i.e. medications for which the indication is not specific to a disease) and underuse of the health care system, among other reasons.^2^-^5^ Misclassification may lead to biased estimates of disease burden: when sensitivity is greater than PPV, prevalence will be overestimated and when sensitivity is less than PPV, prevalence will be underestimated.^6^

Several methods have been proposed to adjust for misclassification bias in prevalence estimates, including correction factors and exit rules. Correction factors for prevalence estimates have been investigated for diabetes,^7^,^8^ breast cancer^9^ and acute myocardial infarction^8^ using sensitivity and specificity estimates obtained from validation studies.^7^,^9^ However, validation studies are often conducted in clinical cohorts with disease prevalence and health-care use patterns that do not reflect those of nonclinical populations,^10^ and the resulting correction factors may not apply to the general population.^7^

Exit rules are deterministic or probabilistic rules used to identify and remove false positives at the individual rather than the population level. Exit rules have been applied in pharmacovigilance and genomics,^11^-^13^ but, to our knowledge, not in the context of chronic disease surveillance using administrative health data. When estimating chronic disease burden, exit rules are well-suited to producing adjusted prevalence estimates that decrease potential overestimation bias that may accumulate over time.^7^,^14^

Peng et al. found that incorporating an exit rule into a hypertension case ascertainment algorithm using a reclassification approach produced results similar to those of a deterministic algorithm.^15^ Whether these findings can be generalized to other chronic diseases is unknown. Given the variability in diagnostic criteria, clinical treatment and population prevalence, further studies to investigate other chronic diseases would be beneficial.

Our purpose was to develop and validate a model-based algorithm that incorporates a reclassification-based exit rule. Our objectives were to validate a logistic regression model-based case ascertainment algorithm; incorporate a reclassification-based exit rule into the algorithm and assess exit-rule performance; and compare prevalence trends for an algorithm with an exit rule to prevalence trends obtained using a previously validated algorithm without an exit rule.

## Methods

***Ethics approval***

Ethics approval was granted by the University of Manitoba’s Health Research Ethics Board (HREB No. HS23961). Data access approval was provided by the Provincial Health Research Privacy Committee (PHRPC No. 2020/2021-12); Manitoba Shared Health with the Winnipeg Regional Health Authority (RAAC2020:026); and Manitoba Primary Care Research Network.

***Study design and data***

We conducted a retrospective cohort study to compare two chronic diseases: diabetes in individuals aged 18 years and younger (referred to as “juvenile diabetes,” or JD, in this article) and multiple sclerosis (MS). 

MS is an immune-mediated disease of the central nervous system in which the protective myelin sheaths around axons and the axons themselves are damaged.^16^ MS onset typically occurs between 20 and 40 years of age.^16^ JD refers to both type 1 and type 2 diabetes diagnosed in individuals aged 18 years and younger. This disease prevents the body from producing insulin or from responding to the insulin it produces, resulting in unregulated blood sugar levels.^17^

Although the etiology of these chronic diseases differ, both are monitored by the Public Health Agency of Canada’s Canadian Chronic Disease Surveillance System (CCDSS), which allows us to compare their algorithm and exit-rule performances.

Study data from 1 April 1995 to 31 March 2022 were obtained from the Manitoba Population Research Data Repository housed at the Manitoba Centre for Health Policy (MCHP) in Winnipeg. Manitoba has a system of universal health care; publicly insured health care services are available for most of the population in the province, and decades of provincial administrative health data have been recorded. These data are ideal for examining the impact of case ascertainment algorithms on chronic disease prevalence trends.

Study cohort sociodemographic information, including health insurance coverage dates, birth dates, sex and residential postal codes, was obtained from the Manitoba Health Insurance Registry. Postal codes from the Registry and average household income from the Statistics Canada census were used to calculate area-level income quintiles.^18^

We obtained the health-care use measures to construct the case ascertainment algorithms and exit rules from the hospital Discharge Abstract Database (DAD), the Medical Claims/Medical Services database and the Drug Program Information Network database. The DAD captures records of all inpatient stays; these were reported using *International Classification of Diseases, 9th Revision, Clinical Modification* (ICD-9-CM) codes until 31 March 2004 and *International Statistical Classification of Diseases, 10th Revision, Canada* (ICD-10-CA) codes from 1April 2004. The Medical Claims/Medical Services database stores ICD-9-CM code records of outpatient visits. The Drug Program Information Network database stores prescription medication dispensation records from community-based pharmacies, using the World Health Organization’s Anatomical Therapeutic Chemical (ATC) codes.^19^

Three databases were used to construct the study cohorts and provide reference standards for identifying MS and JD cases: the Home Care Minimum Data Set (MDS) Assessment database, for MS cases and non-cases; the Diabetes Education Resource for Children and Adolescents (DER-CA) registry database, for JD cases; and the Manitoba Primary Care Research Network (MaPCReN) database, for JD non-cases.

The Home Care MDS Assessment database captures data on home care assessments of and utilization by all individuals receiving home care services delivered by the Winnipeg Regional Health Authority, which serves approximately 60% of Manitoba’s population. The DER-CA registry database stores information on almost all the children in the province diagnosed with type 1 and 2 diabetes who are referred to the DER-CA program. The MaPCReN database stores electronic medical records from a subset of primary care providers (family physicians, nurse practitioners and community pediatricians) across all health regions in Manitoba. All databases can be linked at the individual level using an anonymized personal health identification number.

***Study cohorts***

The study period for the MS cohort was from 1 April 2004 to 31 March 2022 and for the JD cohort was from 1 April 1995 to 31 March 2022. The study periods were based on data availability and access approvals.

MS cohort members met the following inclusion criteria: one or more assessments in the Home Care MDS Assessment database during the study period; an MS assessment field signed by a physician; an MS assessment that could be linked to the Manitoba Health Insurance Registry; no conflicting MS status indications (i.e. multiple assessments suggesting presence or absence of MS status); 20 years or older at time of assessment; and health care coverage on the MS assessment date and continuous health care coverage for at least 2 years (730 days) between the assessment date and cohort entry. This health care coverage requirement ensured that there were sufficient data to determine MS case status (case vs. non-case).

Cohort entry was the start of the MS study period (1 April 2004) or the start of health care coverage, whichever came later. Cohort exit was the end of health care coverage.

MS case status (case vs. non-case) was ascertained from interRAI assessments. This assessment tool is one of a suite of internationally recognized instruments used by clinicians to assess individuals’ health; it has high sensitivity (0.94) and specificity (1.00) for identifying individuals with MS in the home care setting.^20^ An interRAI assessment is required to be able to access home care in Manitoba.

The DER-CA registry database was used to identify JD cases and the MaPCReN database to identify the non-cases. Individuals categorized as JD cases met all of the following inclusion criteria: a record in the DER-CA database with a diagnosis date within the study period (1 April 1995 to 31 March 2022); an assessment that could be linked to the Manitoba Health Insurance Registry; a diagnosis date before their 18th birthday; health care coverage during the study period and before the individual’s 18th birthday; and at least 2 years (730 days) of continuous coverage between their cohort entry and diagnosis dates.

Individuals categorized as JD non-cases met all of the following inclusion criteria: a record in the MaPCReN database within the study period; an assessment that could be linked to the Manitoba Health Insurance Registry; birthdate between 1 April 1990 and 31 March 2018; no diabetes codes in the MaPCReN data and no diabetes listed as a condition list before their 18th birthday; and at least 2 years (730 days) of continuous health care coverage between cohort entry and exit dates. JD case definitions in MaPCReN have high sensitivity (0.97) and specificity (1.00).^21^ Individuals categorized as JD non-cases were also excluded if they had records in the DER-CA database. 

For both JD cases and non-cases, the cohort entry date was the start of the study period (1 April 1995) or the start of health care coverage, whichever came later. To ensure age comparability between cases and controls, individuals were removed from the cohort on their 18th birthday. Therefore, cohort exit was defined as the individual’s last health care coverage date or the date of their 18th birthday, whichever came first.

***Study variables***

Health care use variables included the number of general physician visits, the number of specialist physician visits, any hospitalizations (binary: yes or no) and any disease-specific health care use (i.e. any physician visit or hospitalization with an MS or JD diagnosis code or any MS- or JD-specific prescription medication). These variables were defined for each fiscal year (1 April to 31 March of the following calendar year) during the study period.

For the MS cohort, neurologist visits were excluded from the specialist physician visit count because visits to the provincial MS clinic were not recorded between 2000 and 2010.

MS diagnosis codes were ICD-9-CM 340 and ICD-10-CA G35. JD diagnosis codes were ICD-9-CM 250 and ICD-10-CA E10 to E14. ATC codes for MS- and JD-specific prescription medications are listed in the supplementary material (Table S1; available on request from the authors).

***Algorithm development and statistical analysis***

The sociodemographic characteristics of individuals categorized as cases and non-cases were compared using χ^2^ tests for categorical variables and *t*tests for continuous variables. Logistic regression models fitted to the data for each cohort contained health-care use variables based on 1, 3 and 5 years of data. For the 3-year and 5-year models, case dates were defined as the last year of the period (e.g. for the 3-year model, data from fiscal years 2000 to 2002 were used to define covariates for 2002). To be included in model building and validation, individuals had to have at least one day of health care coverage in each year used to build the model. Model covariates also included sex and income quintile. Residence location was included as a covariate solely for the JD cohort, as the Home Care MDS Assessment database stores data of urban residents only.

To build and validate the model-based case ascertainment algorithms, the MS and JD cohorts were randomly split without stratification into 70% training and 30% validation cohorts. Algorithm building and validation required 1, 3 or 5 years of data, depending on the model; most individuals had more than 5 years of data available. Therefore, the data to build and validate algorithms were randomly selected for each individual (1, 3 or 5 consecutive years). Predicted probability cut points were determined using the top left value of the receiver operating characteristic curve. Algorithm validation metrics included sensitivity, specificity, PPV and NPV, with their respective 95% confidence intervals.

The reclassification-based exit rule involved applying the trained logistic regression model to each year of available study cohort data. This resulted in multiple classifications throughout the study periods—up to 18 MS and 27 JD classifications for the 1-year logistic regression model-based algorithm and 13 MS and 22 JD classifications for the 5-year logistic regression model-based algorithm. By reclassifying individuals numerous times, false positives could be removed from the case group. If an individual did not have health care coverage in a given year, they were not reclassified.

Reclassif﻿ication performance was assessed using the reclassification rate (total number of reclassifications divided by total number of misclassifications); the average number of misclassifications (of individuals misclassified, the average number of misclassifications per individual); and the average time to reclassification (average number of years between misclassification and correct reclassification).

To assess the impact of the exit rule on prevalence trends, we repeatedly applied the model-based algorithm with the highest estimated validity over the study period to estimate disease prevalence. CCDSS algorithms currently used for MS and JD surveillance were also applied to the study data. The CCDSS algorithm for MS requires either one or more hospitalizations or at least five physician claims with an MS diagnosis code within 2 years.^22^,^23^ The CCDSS algorithm for JD requires either one or more hospitalizations or at least two physician claims with a JD diagnosis code within 2 years.^23^ Case dates were defined as the date of the hospital separation record or the last physician claim, whichever came first.

MS and JD prevalence estimates were calculated per 100000 population using the model-based algorithm with an exit rule and the CCDSS algorithm. A single linear regression model was fitted to the annual prevalence estimates of MS and JD to test for differences in slope coefficient estimates between the model-based algorithm with an exit rule and the CCDSS algorithm. The model contained the year and algorithm type (i.e. model-based vs. CCDSS) main effects and their two-way interaction effect (i.e. intercept + year + algorithm type + [year algorithm type]). To account for potential correlation within the model estimates, a statistically significant difference was based on nominal αlevel of .001.

Analyses were performed using statistical packages SAS version 9.4 (SAS Institute Inc., Cary, NC, US) and R version 4.3.0 (R Foundation for Statistical Computing, Vienna, AT).^24^

## Results

The MS cohort included 60228 individuals of whom 608 (1.0%) were cases; the JD cohort included 44125 individuals of whom 2506 (5.7%) were cases (Table 1). Cohort flow charts are included in the supplementary material (available on request from the authors). Individuals categorized as MS cases included a higher percentage of females, were younger at cohort entry and had more years of healthcare coverage than those categorized as MS non-cases.

**Table 1:** Characteristics of MS and JD cohorts, Manitoba, 1995–2022

Variable	Cases	Non-cases	*p* value
**MS**	**n = 608**	**n = 59 620**	NA
Sex, n (%)			
Males	195 (32.1)	22 130 (37.1)	0.0104
Females	413 (67.9)	37 490 (62.9)	0.0104
Period of MS assessment, n (%)			
2004–2009	331 (54.4)	22 691 (38.1)	< 0.0001
2010–2015	148 (24.3)	19 182 (32.2)	< 0.0001
2016–2021	129 (21.2)	17 747 (29.8)	< 0.0001
Income quintile,^a^ n (%)			
Q1 (lowest)	101 (16.6)	14 245 (23.0)	< 0.0001
Q2	122 (20.1)	13 096 (22.0)	< 0.0001
Q3	125 (20.6)	12 112 (20.3)	< 0.0001
Q4	112 (18.4)	10 156 (17.0)	< 0.0001
Q5 (highest)	142 (23.4)	9542 (16.0)	< 0.0001
Age at cohort entry, mean (SD) years	54.2 (12.8)	69.4 (13.2)	< 0.0001
Age at assessment, mean (SD) years	61.2 (12.7)	77.9 (12.3)	< 0.0001
Total health care coverage, mean (SD) years	15.0 (4.2)	13.3 (4.7)	< 0.0001
Health care coverage before assessment, mean (SD) years	7.0 (4.9)	8.5 (4.9)	< 0.0001
Health care coverage after assessment, mean (SD) years	8.0 (5.0)	4.8 (3.8)	< 0.0001
**JD^b^**	**n = 2506**	**n = 41 619**	NA
Sex, n (%)			
Males	1234 (49.2)	20 710 (49.8)	0.6137
Females	1272 (50.8)	20 909 (50.2)	0.6137
Period of cohort entry, n (%)			
1995–2000	1483 (59.2)	11 722 (28.2)	< 0.0001
2001–2007	744 (29.7)	9816 (23.6)	< 0.0001
2008–2013	246 (9.8)	10 104 (24.3)	< 0.0001
2014–2019	33 (1.3)	9977 (24.0)	< 0.0001
Income quintile,^c^ n (%)			
Q1 (lowest)	915 (36.6)	6647 (16.2)	< 0.0001
Q2	511 (20.5)	6823 (16.6)	< 0.0001
Q3	350 (14.0)	8707 (21.2)	< 0.0001
Q4	390 (15.6)	10 681 (26.0)	< 0.0001
Q5 (highest)	331 (13.3)	8217 (20.0)	< 0.0001
Age at cohort entry, mean (SD) years	1.8 (3.1)	0.9 (2.4)	< 0.0001
Age at diagnosis, mean (SD) years	11.2 (3.7)	NA	NA
Total health care coverage, mean (SD) years	15.0 (3.6)	12.1 (5.1)	< 0.0001

**Source: **Administrative health data from 1995 to 2022 obtained from the Manitoba Population Research Data Repository, Manitoba Centre for Health Policy, Winnipeg, MB. 

**Abbreviations:** JD, juvenile diabetes; MS, multiple sclerosis; NA, not applicable; Q, quintile; SD, standard deviation. 

^a^ 371 cases in the MS cohort had missing income quintile information. 

^b^ JD (“juvenile diabetes”) refers to both type 1 and type 2 diabetes in individuals aged 18 years and younger. 

^c^ 553 cases in the JD cohort had missing income quintile information. 

Individuals categorized as JD cases were older at cohort entry and had more years of health care coverage than those classified as non-cases (Table 1).

For the MS cohort, “any MS-specific health care use” was the only covariate that was statistically significant across multiple models (Table 2). Specialist and general physician visits were statistically significant covariates in the 1- and 5-year models, respectively. Income quintile at baseline was statistically significant for the 1- and 3-year models, but not the 5-year model.

**Table 2:** Logistic regression model odds for 1-, 3- and 5-year algorithms for the MS and JD cohorts, Manitoba, 1995–2022

Predictor	OR (95% CI)		
	1 year	3 years	5 years
MS			
General physician visit(s)	0.99 (0.98–1.01)	0.99 (0.99–1.00)	0.99 (0.99–1.00)^*^
Specialist physician visit(s)	0.98 (0.96–0.99)^*^	1.00 (0.99–1.01)	1.00 (1.00–1.00)
Any hospitalization	1.05 (0.74–1.49)	0.78 (0.6–1.01)	0.87 (0.72–1.04)
Any MS-specific health care use	Not estimated	114.48 (80.25–163.29)^*^	25.85 (20.44–32.70)^*^
Sex (female vs. male)	1.12 (0.83–1.52)	1.11 (0.76–1.64)	1.27 (0.84–1.90)
Income quintile			
Missing vs. Q5 (highest)	0.07 (0.01–0.35)^*^	0.09 (0.01–0.67)^*^	0.02 (<0.001–2.84)
Q1 (lowest) vs. Q5	0.44 (0.28–0.69)^*^	0.40 (0.22–0.73)^*^	0.56 (0.31–1.03)
Q2 vs. Q5	0.39 (0.24–0.61)^*^	0.42 (0.23–0.76)^*^	0.56 (0.3–1.03)
Q3 vs. Q5	0.71 (0.46–1.08)	0.81 (0.49–1.36)	0.98 (0.56–1.71)
Q4 vs. Q5	0.63 (0.40–0.99)^*^	0.80 (0.46–1.39)	0.94 (0.52–1.70)
JD^a^			
General physician visit(s)	0.95 (0.90–0.99)^*^	0.99 (0.97–1.00)	0.98 (0.97–1.00)^*^
Specialist physician visit(s)	1.05 (1.04–1.07)^*^	1.02 (1.01–1.03)^*^	1.01 (1.01–1.02)^*^
Any hospitalization	0.81 (0.52–1.26)	1.37 (1.06–1.77)^*^	1.30 (1.07–1.58)^*^
Any JD-specific health care use	Not estimated	559.83 (424.33–738.59)^*^	271.08 (209.78–350.3)^*^
Sex (female vs. male)	1.19 (0.93–1.52)	1.00 (0.76–1.30)	0.91 (0.70–1.18)
Income quintile			
Missing vs. Q5 (highest)	0.46 (0.05–4.08)	0.52 (0.05–5.26)	2.06 (0.17–25.06)
Q1 (lowest) vs. Q5	6.86 (4.43–10.63)^*^	4.57 (2.96–7.05)^*^	4.02 (2.60–6.22)^*^
Q2 vs. Q5	2.84 (1.76–4.59)^*^	2.42 (1.51–3.88)^*^	2.87 (1.81–4.54)^*^
Q3 vs. Q5	1.69 (1.03–2.79)^*^	1.28 (0.78–2.11)	1.36 (0.84–2.21)
Q4 vs. Q5	0.94 (0.57–1.57)	0.94 (0.58–1.52)	1.02 (0.63–1.66)
Residence location (rural vs. urban)	1.51 (1.16–1.95)^*^	1.24 (0.94–1.63)	1.12 (0.86–1.45)

**Source:** Administrative health data from 1995 to 2022 obtained from the Manitoba Population Research Data Repository, Manitoba Centre for Health Policy, Winnipeg, MB. 

**Abbreviations:** CI, confidence interval; JD, juvenile diabetes; MS, multiple sclerosis; OR, odds ratio; Q, quintile. 

^a^ JD (“juvenile diabetes”) refers to both type 1 and type 2 diabetes in individuals aged 18 years and younger. 

* Odds ratios are statistically significant. 

In the JD cohort, specialist physician visit, any JD-specific health care use, income quintile 1 and income quintile 2 were statistically significant covariates in all three models ( Table 2).

For MS, all algorithms demonstrated high specificity and NPV (Table 3). Sensitivity was lowest for the 1-year algorithm (0.62) and highest for the 5-year algorithm (0.85). The 3-year algorithm had sensitivity (0.82) similar to the 5-year algorithm (0.85) and the highest PPV (0.60).

**Table 3:** Validity estimates for model-based algorithms using 1, 3 and 5 years of administrative health data for MS and JD, Manitoba, 1995–2022

Measure	1 year	3 years	5 years
MS			
Sensitivity	0.62	0.82	0.85
Specificity	0.98	0.99	0.99
PPV	0.21	0.60	0.44
NPV	1.00	1.00	1.00
JD^a^			
Sensitivity	0.87	0.94	0.95
Specificity	1.00	1.00	0.99
PPV	0.95	0.93	0.92
NPV	0.99	1.00	1.00

**Source:** Administrative health data from 1995 to 2022 obtained from the Manitoba Population Research Data Repository, Manitoba Centre for Health Policy, Winnipeg, MB. 

**Abbreviations:** JD, juvenile diabetes; MS, multiple sclerosis; NPV, negative predictive value; PPV, positive predictive value. 

^a^ JD (“juvenile diabetes”) refers to both type 1 and type 2 diabetes in individuals aged 18 years and younger. 

For JD, all the algorithms had high specificity and NPV (Table 3). The 5-year algorithm had the highest sensitivity (0.95) and the 1-year algorithm had the highest PPV (0.95).

In the MS cohort, the 1-year algorithm with an exit rule had the highest reclassification rate for non-cases (0.33) and cases (0.28) (Table 4). Rates were similar for the remaining two algorithms with an exit rule (0.22 for non-cases; 0.14–0.18 for cases). The average number of misclassifications was higher for MS cases than non-cases. The average was similar for all algorithms with exit rules for MS non-cases (3.73–3.77) and more variable across algorithms for MS cases (4.82–6.68). For both MS cases and non-cases, the 1-year algorithm with an exit rule had the lowest time to reclassification (cases: 2.50 years; non-cases: 2.08 years).

**Table 4:** Reclassification performance for model-based algorithms using 1, 3 and 5 years of administrative health data stratified by the reference standard-based MS and JD non-cases and cases, Manitoba, 1995–2022

Performance measure	Non-cases			Cases		
	1 year	3 years	5 years	1 year	3 years	5 years
MS						
Number of misclassifications, n	6310	1170	2151	1068	489	270
Number of reclassifications, n	2053	263	476	294	88	39
Reclassification rate^a^	0.33	0.22	0.22	0.28	0.18	0.14
Average number of misclassifications per individual, n	3.75	3.73	3.77	6.68	5.37	4.82
Time to reclassification, years	2.08	3.09	3.58	2.50	3.51	3.03
JD^b^						
Number of misclassifications, n	484	549	586	585	280	252
Number of reclassifications, n	313	161	105	74	40	39
Reclassification rate^a^	0.65	0.29	0.18	0.13	0.14	0.15
Average number of misclassifications per individual, n	1.71	2.76	3.51	2.88	3.08	3.50
Time to reclassification, years	1.45	2.58	3.43	1.47	1.35	1.51

**Source:** Administrative health data from 1995 to 2022 obtained from the Manitoba Population Research Data Repository, Manitoba Centre for Health Policy, Winnipeg, MB. 

**Abbreviations:** JD, juvenile diabetes; MS, multiple sclerosis. 

^a^ Total number of reclassifications divided by total number of misclassifications. 

^b^ JD (“juvenile diabetes”) refers to both type 1 and type 2 diabetes in individuals aged 18 years and younger. 

In the JD cohort, non-cases had higher reclassification rates and greater variability across the 1-, 3- and 5-year algorithms (0.18–0.65) than cases (0.13–0.15) ( Table 4). JD non-cases also had a lower average number of misclassifications compared to cases; the 1-year algorithm with an exit rule showed the lowest average number of misclassifications per individual for both non-cases (1.71) and cases (2.88). The time to case reclassification varied from 1.45 to 3.43 years for JD non-cases and from 1.35 to 1.51 years for JD cases; the 1-year and 3-year algorithms with an exit rule had the shortest time to reclassification for non-cases and cases, respectively.

**Figure 1:**
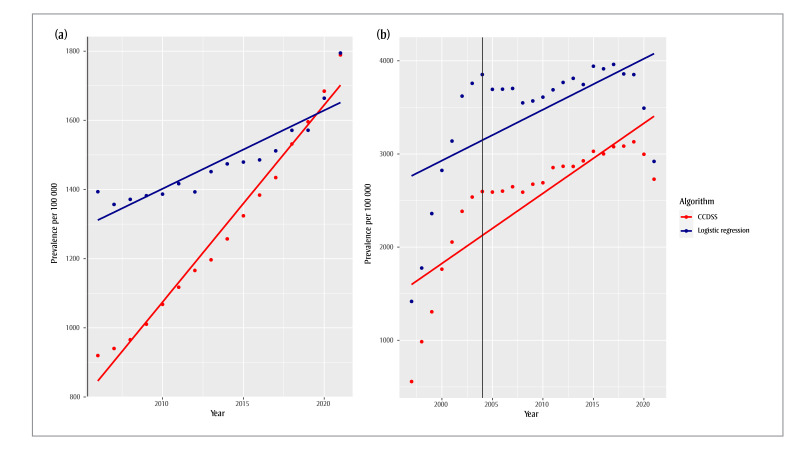
Prevalence trends for the CCDSS algorithm and the 3-year model-based algorithm with an exit rule for (a) MS and (b) JDa, Manitoba, 1995–2022

**Abbreviations:** CCDSS, Canadian Chronic Disease Surveillance System; ICD-9-CM, *International Classification of Diseases, 9th Revision, Clinical Modification; ICD-10-CA, International Statistical Classification of Diseases, 10th Revision, Canada*; JD, juvenile diabetes; MS, multiple sclerosis. 

**Note:** The change from ICD-9-CM to ICD-10-CA coding in the Discharge Abstract Database on 1 April 2004 is indicated by the solid black vertical line. 

^a^ JD (“juvenile diabetes”) refers to both type 1 and type 2 diabetes in individuals aged 18 years and younger. 

For both MS and JD, the 3-year model-based algorithm with an exit rule was selected for comparison with the CCDSS algorithm. The model-based algorithm with an exit rule calculated a higher prevalence than the CCDSS algorithm for MS and JD (Table 5). When assessing differences in slopes between algorithms (i.e. whether the change in population health is similar across algorithms), there was a statistically significant difference in the slopes for MS, where the model-based algorithm with an exit rule had a lower slope (i.e. slower increase in prevalence) compared to the CCDSS algorithm. There was no difference in slopes for JD. Model parameters are reported in Table 5 and prevalence trends for each algorithm are in Figure 1.

**Table 5:** Linear regression model parameter estimates and fit statistics comparing prevalence trends from the best-performing model-based algorithm with an exit rule and the CCDSS algorithm for MS and JD, Manitoba, 1995–2022

Predictor/statistic	Estimate (SE)
MS	
Intercept	846.38 (23.21)^*^
Year	57.01 (2.64)^*^
Algorithm (ref: CCDSS algorithm)	465.08 (32.83)^*^
Year algorithm	−34.34 (3.73)^*^
Model fit: *R*^2^	0.96
JD^a^	
Intercept	1598.74 (184.83)^*^
Year	75.24 (13.2)^*^
Algorithm (ref: CCDSS algorithm)	1166 (261.38)^*^
Year algorithm	−20.58 (18.67)
Model fit: *R*^2^	0.68

**Source:** Administrative health data from 1995 to 2022 obtained from the Manitoba Population Research Data Repository, Manitoba Centre for Health Policy, Winnipeg, MB. 

**Abbreviations:** CCDSS, Canadian Chronic Disease Surveillance System; JD, juvenile diabetes; MS, multiple sclerosis; ref, reference; SE, standard error. 

^a^ JD (“juvenile diabetes”) refers to both type 1 and type 2 diabetes in individuals aged 18 years and younger. 

* *p* < 0.001. 

## Discussion

The 3-year algorithm performed best for both MS and JD, with disease-specific health care use the primary predictor of disease status. Exit rules reclassified both MS and JD non-cases at a higher rate than the respective cases. The 1-year algorithm resulted in the shortest time to reclassification, irrespective of disease. When we compared the prevalence trend of the best-performing (3-year) logistic model-based algorithm with that of the disease-specific CCDSS algorithm, we only observed differences for MS, with the model-based algorithm with an exit rule having a lower slope (i.e. a slower increase in prevalence) than the CCDSS algorithm. 

Previous validation studies of the CCDSS MS algorithm reported a sensitivity of 0.84 and a PPV of 0.86.^22^ Nakhla et al. estimated the sensitivity of the CCDSS JD algorithm to be 0.98 and the PPV to be 0.79.^25^ We estimated similar sensitivity in this study (0.82 for MS and 0.94 for JD based on the 3-year logistic regression algorithms). In contrast, we estimated the PPV for MS to be lower (0.60) and the PPV for JD to be higher (0.93) than that reported for the CCDSS algorithm.^25^ The lower PPV for MS may be because we used a home care–based cohort of patients with MS; these patients can have higher rates of MS-adjacent conditions than the general population, making it difficult to differentiate between cases and non-cases. The lower PPV could also be due to our excluding codes from neurologist visits, often the source of MS-specific care. Both cohorts had low prevalence of disease (1% for MS; 6% for JD), which likely contributed to the observed low PPV and high NPV.

Previous research found no difference in prevalence trend estimates for hypertension when using an algorithm without an exit rule compared to an algorithm with a reclassification-based exit rule.^15^ In contrast, we found prevalence trends for JD, but not for MS, to be similar when using the model-based algorithm with an exit rule and the CCDSS algorithm without an exit rule. For MS, differences in slope may result from the exit rule reclassifying false positives and reducing bias. Of note, MS had a lower PPV across all model-based algorithms compared to JD, suggesting that this algorithm may be susceptible to false-positive build-up in the absence of an exit rule. Alternatively, the slower increase in prevalence may be due to individuals not seeking MS-related care during periods of remission or due to MS-specific visits to neurologists not being captured, resulting in their exclusion from the case cohort.

***Strengths and limitations***

The MS study period began after the change in ICD versions used in the DAD, in 2004. Extending the MS study period to include data from before 1 April 2004 may influence the MS prevalence trends calculated using either the model-based algorithm with an exit rule or the CCDSS algorithm due to changes in clinical practice and direct changes to the codes themselves. However, given that the ICD-9-CM and ICD-10-CA diagnosis codes for MS convey similar levels of diagnostic specificity, the effect of the version change is likely minimal. Moreover, research validating MS case definitions (without exit rules) that covered the period before and after 2004 found no changes in disease incidence (a key contributor to prevalence estimates) despite the changes in ICD codes and diagnostic criteria.^26^,^27^

A strength of this study is the use of model-based case ascertainment algorithms, which are known to perform better than deterministic algorithms.^28^ We used several methods to assess algorithm and exit-rule performance—common validation metrics (sensitivity, specificity, PPV, NPV); reclassification rates and time to reclassification stratified by case status; and prevalence trend comparisons with the CCDSS algorithm. Moreover, we evaluated the performance of the algorithm plus exit rule with two diseases with different presentations, diagnostic procedures and affected populations, resulting in a comprehensive understanding of its application in estimating population health.

Limitations of this study include the use of a home care–based treatment population to define MS cases and non-cases. This may limit generalizability of the findings as this population tends to have greater health care needs than the general population. However, use of this cohort provided reasonable indication of MS status.

## Conclusion

Case ascertainment algorithms that incorporate an exit rule can reduce overestimation bias by allowing misclassified non-cases to be correctly reclassified at a later time. The advantages of this approach benefits diseases with low PPV, such as MS, more than the diseases with high sensitivity and PPV due to specific diagnostic codes, such as JD.

## Availability of data and materials

The data used in this article were derived from administrative health data as secondary use. The data were provided to the investigators under specific data-sharing agreements and were only for approved use at the MCHP. The original source data are not owned by the researchers or MCHP and cannot be shared through a public repository. Approval for use of the original data is noted in the “Ethics approval ” section. Where necessary, source data specific to this article or project may be reviewed at MCHP with the consent of the original data providers and the required privacy and ethical review bodies.

## Acknowledgements

NCH was supported by the Visual and Automated Disease Analytics (VADA) Program at the time of completing this research.

RAM receives funding from the Canadian Institutes of Health Research (CIHR), MS Canada, the National Multiple Sclerosis Society, the Consortium of Multiple Sclerosis Centers, Brain Canada, Arthritis Society Canada, the US Department of War and Pfizer Foundation. RAM is supported by the Multiple Sclerosis Clinical Research Chair in the Faculty of Medicine at Dalhousie University.

LML is a Tier 1 Canada Research Chair (CRC-2023-00349) and receives funding from the CIHR.

## Conflicts of interest

Authors have no competing interests to declare. RAM is a co-investigator on a study funded in part by Biogen Idec and Roche Canada, but neither RAM nor her institution receive any funds from these organizations.

## Funding

Funding support for this research was provided by the CIHR (Funding Reference No. FDN 143293).

## Authors’ contributions and statement

NCH: Conceptualization, formal analysis, methodology, writing—original draft, writing—review and editing.

RAM: Conceptualization, methodology, writing—review and editing.

DJ: Conceptualization, methodology, writing—review and editing.

PI: Conceptualization, methodology, writing—review and editing.

LLM: Conceptualization, funding acquisition, methodology, formal analysis, writing—review and editing, supervision.

All authors approved the final manuscript.

The content and views expressed in this article are those of the authors and do not necessarily reflect those of the Government of Canada. Additionally, no official endorsement by the Manitoba Centre for Health Policy, Manitoba Health, or other data providers is intended or should be inferred.
